# Highly diversified Zika viruses imported to China, 2016

**DOI:** 10.1007/s13238-016-0274-5

**Published:** 2016-05-21

**Authors:** Yanjun Zhang, Wenxian Chen, Gary Wong, Yuhai Bi, Juying Yan, Yi Sun, Enfu Chen, Hao Yan, Xiuyu Lou, Haiyan Mao, Shichang Xia, George F. Gao, Weifeng Shi, Zhiping Chen

**Affiliations:** Zhejiang Provincial Center for Disease Control and Prevention, Hangzhou, 310051 China; Yiwu Center for Disease Control and Prevention, Yiwu, 322000 China; CAS Key Laboratory of Pathogenic Microbiology and Immunology (CASPMI), Institute of Microbiology, Chinese Academy of Sciences, Beijing, 100101 China; Institute of Pathogen Biology, Taishan Medical College, Taian, 271000 China; Center for Influenza Research and Early-Warning (CASCIRE), Chinese Academy of Sciences, Beijing, 100101 China; Shenzhen Key Laboratory of Pathogen and Immunity, Shenzhen Third People’s Hospital, Shenzhen, 518112 China; Office of Director-General, Chinese Center for Disease Control and Prevention (China CDC), Beijing, 102206 China

**Dear Editor,**

First discovered during 1947 in Uganda from febrile rhesus macaques, Zika virus (ZIKV) is a mosquito-borne, re-emerging flavivirus historically known to be present in much of Africa and Asia, occasionally causing outbreaks amongst the local populace (Haddow et al., 2012). ZIKV infections in humans are mostly asymptomatic, but a small percentage of patients may show clinical symptoms such as a fever and rash, which resolve within a week or less. Due to the benign nature of the disease, ZIKV was considered an obscure and neglected pathogen of low public health consequence. Recently, viral infection of women during pregnancy have been associated with microcephaly in their offspring (Ventura et al., 2016), and neurological disorders such as Guillain-Barré syndrome (GBS) have also been associated with prior ZIKV infections (Cao-Lormeau et al., 2016). Moreover, the ZIKV nonstructural protein 1 (NS1) has diverse electrostatic characteristics at host-interaction interfaces (Song et al., 2016). These new findings, in addition to the persistence of ZIKV in the semen of infected patients (Lazear and Diamond 2016) and possible sexual transmission of the virus (Oster et al., 2016) suggest that this virus may be more dangerous than initially thought.

ZIKV first attracted global attention during 2007 when it caused an outbreak in Micronesia (Duffy et al., 2009), before spreading through Oceania in subsequent years (Cao-Lormeau et al., 2014; Dupont-Rouzeyrol et al., 2015). ZIKV eventually arrived in South America and was identified in Brazil during 2015 (Campos et al., 2015), and rapidly spread throughout the continent as well as the Caribbean islands. As of March 22nd, 2016, a total of 4,800 laboratory-confirmed, natural ZIKV infections have been reported over 46 countries, and an additional 650 imported cases have been reported by 32 more countries from Europe, Asia and North America.

*Aedes aegypti* and *Aedes albopictus* are the two major vectors for ZIKV transmission. In China, *Aedes aegypti* is mostly distributed in Hainan, southern Guangdong and Guangxi, whereas *Aedes albopictus* is widespread in southern and central China (Kraemer et al., 2015). As of April 5th, 2016, a total of 13 ZIKV cases have been imported into from travelers since the first patient was reported on February 5th, 2016, and all cases have had prior travel history to South America or Oceania (Table 1). Nine travelers returned from Venezuela, one from Suriname and three from Fiji/Samoa. Five arrived in Hong Kong first and then entered mainland China via Shenzhen in Guangdong Province, seven entered Guangdong Province directly via Guangzhou, and one entered mainland China through Shanghai. Six cases showed mild clinical symptoms (i.e. rash and/or fever) upon arrival at Chinese customs (Fig. 1A), and four were detected by customs staff (Table 1). The travelers who were asymptomatic at customs developed symptoms 2–9 days after returning to China (Table 1). Other potential symptoms from ZIKV disease include conjunctivitis, which was observed in case #3 (Fig. 1B). Due to the high density of *Aedes aegypti* and *Aedes albopictus* mosquitoes in southern China and that the majority of cases entered mainland China through Guangdong Province, we believe southern China is especially at risk for more ZIKV infections.

**Table 1:** **Summary of imported ZIKV cases into China, February to March 2016.**

**Case No.**	**Date of entry into China**	**Prior travel history**	**Point of entry into mainland China**	**Clinical symptoms at customs?**	**Detected by customs?**	**Date of symptom onset after entry into mainland China**	**Hospitalization location**	**Date reported by China**
1	05-Feb-16	Venezuela	Hong Kong-Shenzhen	Yes	No		Ganzhou, Jiangxi Province	09-Feb-16
2	09-Feb-16	Venezuela	Guangzhou	Yes	Yes		Guangzhou, Guangdong Province	15-Feb-16
3	14-Feb-16	Fiji/Samoa	Hong Kong-Shenzhen	Yes	Yes		Yiwu, Zhejiang Province	19-Feb-16
4	15-Feb-16	Fiji/Samoa	Hong Kong-Shenzhen	No	No	20-Feb-16	Yiwu, Zhejiang Province	24-Feb-16
5	15-Feb-16	Fiji/Samoa	Hong Kong-Shenzhen	No	No	21-Feb-16	Yiwu, Zhejiang Province	24-Feb-16
6	21-Feb-16	Suriname	Shanghai	Yes	No		Wenzhou, Zhejiang Province	26-Feb-16
7	25-Feb-16	Venezuela	Guangzhou	Yes	Yes		Guangzhou, Guangdong Province	27-Feb-16
8	25-Feb-16	Venezuela	Guangzhou	Yes	Yes		Guangzhou, Guangdong Province	27-Feb-16
9	19-Feb-16	Venezuela	Guangzhou	No	No	26-Feb-16	Enping, Guangdong Province	29-Feb-16
10	25-Feb-16	Venezuela	Guangzhou	No	No	29-Feb-16	Enping, Guangdong Province	02-Mar-16
11	03-Mar-16	Venezuela	Guangzhou	No	No	05-Mar-16	Enping, Guangdong Province	07-Mar-16
12	03-Mar-16	Venezuela	Guangzhou	No	No	05-Mar-16	Enping, Guangdong Province	07-Mar-16
13	29-Feb-16	Venezuela	Hong Kong-Shenzhen	No	No	09-Mar-16	Enping, Guangdong Province	11-Mar-16

**Figure 1:**
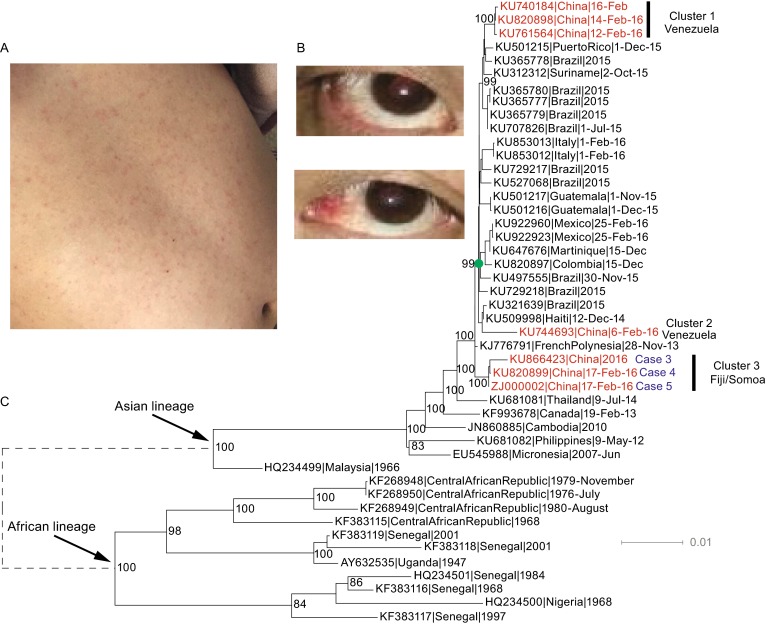
Symptoms of ZIKV disease and phylogenetic tree of full-length ZIKV genome sequences. (A) Rash and (B) conjunctivitis are readily observed in the patient. (C) The Maximum likelihood tree estimated using full-length ZIKV genome sequences. The seven available sequences from imported cases to China are highlighted in red.

Seven full-length genome sequences were analyzed in this study, two of which (cases #4 and #5) were sequenced by our group. Cases #4 and #5 are father and son, and were admitted into hospital in Yiwu, Zhejiang Province on February 20th and 21st, respectively. Thirty-nine additional complete ZIKV genome sequences, including those from Africa, Asia as well as North/Central/South America were obtained from GenBank. Multiple sequence alignment was performed using Muscle (Edgar 2004) and the maximum likelihood phylogenetic tree was constructed using RAxML (Stamatakis 2014). One thousand bootstrap replicates were run and the GTRGAMMA model was applied.

Phylogenetic analysis of the full-length ZIKV genome sequences confirmed the proposed classification of ZIKV into two major lineages (Enfissi et al., 2016), African and Asian, and sequences from the current outbreak fell within the Asian lineage (Fig. 1C). Consistent with a recent report (Faria et al., 2016), our results also suggested a single introduction of ZIKV into South America (Fig. 1C). Based on the current surveillance data, the geographical source of ZIKV transmission into South America is still unclear, but French Polynesia is thought to be one of the most likely source regions (Faria et al., 2016) (Fig. 1C).

Interestingly, our results showed that the seven Chinese imported cases did not cluster together; rather, they formed three independent clusters. Cluster 1 included three isolates from Venezuela. Cluster 2 contained one isolate also from Venezuela, but Clusters 1 and 2 do not group together despite originating from the same country. Cluster 3 was composed of three isolates from Zhejiang Province (cases #3–5), all of whom returned from Fiji/Somoa. The sequence divergence between Clusters 1 & 2 was 0.8% ± 0.1%, and those between Clusters 1 & 3 and between Clusters 2 & 3 were 0.7% ± 0.1%, 1.0% ± 0.1%, respectively. However, the within group divergence of Clusters 1 and 3 were only 0.1% and 0.2%, respectively. Therefore, the between group sequence divergences of the three clusters were much higher than the within group divergences, also supporting the classification of the seven available Chinese ZIKV sequences into three clusters.

We then studied the amino acid polymorphisms of ZIKV across the three clusters (Fig. S1). First, Cluster 2 had distinct amino acid polymorphisms from Clusters 1 and 3. Second, the amino acid polymorphisms were different between Clusters 1 and 3 at certain positions, such as residue 109 of the C protein, residue 419 of the E protein, and residue 328 of the NS1 protein. Third, there were also several cluster-specific amino acid signatures. For instance, Ile233 and Ala271 of the NS5 protein were specific for Cluster 1, whereas Asn109 of the C protein, Arg419 of the E protein, Ser628 and Arg674 of the NS5 protein were specific for Cluster 3.

In summary, our analysis demonstrates a high genetic diversity of ZIKV from cases imported into China, and suggests that ZIKV may have diversified phylogenetically in the outbreak areas (Shi et al., 2016). Our data indicates an urgent need to investigate ZIKV evolution in nature and to assess for the biological significance of these mutations.

## Electronic supplementary material

Below is the link to the electronic supplementary material.

Supplementary material 1 (PDF 368 kb)
