# Supplementary material for: Highly diversified Zika viruses imported to China, 2016
Source: Protein Cell. 2016 May 21;7(6):461–4. doi: 10.1007/s13238-016-0274-5 (PMC4887330; doi:10.1007/s13238-016-0274-5)
Supplement: Supplementary file 1 — Supplementary material 1 (PDF 368 kb) [file 13238_2016_274_MOESM1_ESM.pdf]

	C			E									
	76	107	109	323	442	445	503	520	612	613	620	623	
KJ776791@2013-11-28	E	D	S	V	I	D	V	D	L	H	V	A	
KU740184@2016-02	.	E	.	.	.	G	.	.	.	.	.	.	Cluster 1
KU820898@2016-02-14	.	E	.	.	.	.	.	.	.	.	.	.	
KU761564@2016-02-12	.	.	.	.	.	.	.	.	.	.	.	.	
KU744693@2016-02-06	D	.	.	A	L	.	A	A	V	D	G	G	Cluster 2
KU820899@2016-02-17	.	.	N	.	.	.	.	.	.	.	.	.	Cluster 3
ZJ000002@2016-02-17	.	.	N	.	.	.	.	.	.	.	.	.	
KU866423@2016	.	.	N	.	.	.	.	.	.	.	.	.	
	76	107	109	33	152	155	213	230	322	323	330	333	

E		NS1							NS3					NS4B			
709	739	794	795	970	1005	1050	1107	1118	1856	1857	1867	1938	2027	2295	2313	2317	
K	F	A	D	S	R	T	C	R	D	H	S	D	R	I	A	T	
.	.	.	.	.	.	.	.	.	.	.	.	.	.	.	.	.	Cluster 1
.	.	.	.	.	.	.	.	.	.	.	.	.	.	.	.	.	
.	.	.	.	.	.	.	.	.	.	.	.	.	.	.	.	.	
.	I	G	G	W	W	A	S	Q	E	Y	R	G	.	M	P	S	Cluster 2
R	.	.	.	.	.	.	.	Q	.	.	.	.	.	.	.	.	Cluster 3
R	.	.	.	.	.	.	.	Q	.	.	.	.	.	.	.	.	
R	.	.	.	.	.	.	.	Q	.	.	.	.	K	.	.	.	
419	449	4	5	180	215	260	317	328	358	359	369	440	529	30	48	52	

NS4B		NS5															
2419	2445	2634	2644	2749	2787	2807	2809	2831	2833	2974	2975	3144	3190	3334	3392	3403	
D	I	M	V	T	V	S	H	E	P	N	M	N	K	V	V	M	
.	.	V	.	I	A	.	.	.	.	.	.	.	.	.	.	.	Cluster 1
.	.	V	.	I	A	.	.	.	.	.	.	.	.	.	.	.	
.	.	V	.	I	A	.	.	.	.	.	.	.	.	.	.	.	
E	M	V	.	.	.	A	K	D	A	I	T	.	.	A	.	.	Cluster 2
.	.	.	.	.	.	.	.	.	.	.	.	S	R	.	.	.	Cluster 3
.	.	.	.	.	.	.	.	.	.	.	.	S	R	.	.	.	
.	.	.	I	.	.	.	.	.	.	.	.	S	R	.	M	V	
154	180	118	128	233	271	291	293	315	317	458	459	628	674	818	876	887	

Figure S1. Distinct amino acid polymorphisms of the three ZIKV clusters. The numbers at the top indicate the positions with different amino acids in the ZIKV genomes, and the numbers at the bottom indicate the positions with different amino acids in each of the ZIKV encoded proteins.
